# Efficacy and safety of platelet-rich plasma and autologous-serum eye drops for dry eye in primary Sjögren’s syndrome: a randomized trial

**DOI:** 10.1038/s41598-023-46671-2

**Published:** 2023-11-07

**Authors:** Min-Ji Kang, Jee Hye Lee, Jehyung Hwang, So-Hyang Chung

**Affiliations:** 1 Department of Ophthalmology, College of Medicine, Sanggye Paik Hospital, Inje University, Seoul, Republic of Korea; 2 Department of Ophthalmology, College of Medicine, Seoul St. Mary’s Hospital, The Catholic University of Korea, #222 Banpo-daero, Seocho-gu, Seoul, 06591 Republic of Korea

**Keywords:** Corneal diseases, Diseases, Eye diseases, Conjunctival diseases

## Abstract

We compared the efficacy and safety of autologous-serum (AS) and platelet-rich plasma (PRP) eye drops for dry eye (DE) treatment in primary Sjögren’s syndrome (SS). This prospective, randomized, double-blinded clinical study included patients diagnosed with primary SS DE. Thirty-eight participants were randomly assigned to the AS or PRP groups. Corneal and conjunctival staining scores, Schirmer I test, tear film break-up time (TBUT), and ocular surface disease index (OSDI) scores were evaluated at 4 and 12 weeks. Conjunctival impression cytology (CIC) metaplasia grade and goblet cell density grade at 12 weeks were compared with those at baseline. Corneal and conjunctival staining scores and TBUT significantly improved at 4 and 12 weeks in both groups (all p < 0.005). No significant difference between the AS and PRP groups was observed at 4 and 12 weeks. The Schirmer I values, OSDI scores, CIC metaplasia grade, and goblet cell density grade did not significantly change at 4 and 12 weeks in either group. Both AS and PRP eye drops are effective for primary SS DE without a significant difference. Considering that the preparation time of PRP is shorter than that of AS, PRP can be a good alternative treatment for primary SS DE.

## Introduction

Artificial tears, secretagogues, and anti-inflammatory eye drops have been widely used for dry eye (DE) treatment^1^. Fox et al.^2^ showed that autologous serum (AS) eye drops are useful for the treatment of intractable DE. The composition of AS is similar to that of tears in terms of pH; osmolarity; and levels of vitamin A, immunoglobulin A, and growth factor^3^, which have epitheliotropic potentials and help epithelial healing in ocular surface disorders^4^. Primary Sjögren’s syndrome (SS) is an autoimmune disease that causes the destruction of exocrine glands, such as the lacrimal glands, without any associated systemic disease^5,6^. SS can induce severe chronic DE due to infiltration of lymphocytes in the lacrimal glands and conjunctiva^7^. AS has been reported to have a beneficial effect on symptoms, ocular surface parameters, and confocal microscopic findings in primary SS DE^8,9^.

Platelet-derived eye drops, such as those containing platelet-rich plasma (PRP), plasma rich in growth factors, and platelet lysate, have recently been introduced for treating ocular surface disorders^10–13^. As platelets play a key role in wound healing owing to the high growth factor and cytokine levels^14^, various platelet-derived preparations have been used in regenerative medicine and orthopedic and maxillofacial surgeries^15^. Among them, PRP has been more frequently used than other platelet-derived preparations in eye drops for ocular surface disorders^13,16–19^. Kim et al.^19^ reported higher epidermal growth factor (EGF) levels in PRP than in AS and a higher success rate with PRP than with AS for persistent epithelial defects. Metheetrairut et al.^20^ reported significantly higher transforming growth factor (TGF)-β and fibronectin levels in PRP than in AS; however, AS and PRP showed similar effects on ocular surface signs in non-SS DE.

The clinical effect of AS and PRP in primary SS DE has not been extensively evaluated. This prospective randomized study aimed to compare the efficacy of AS and PRP eye drops on clinical DE parameters and goblet cells in primary SS DE.

## Results

### Patient enrolment

**Figure 1:**
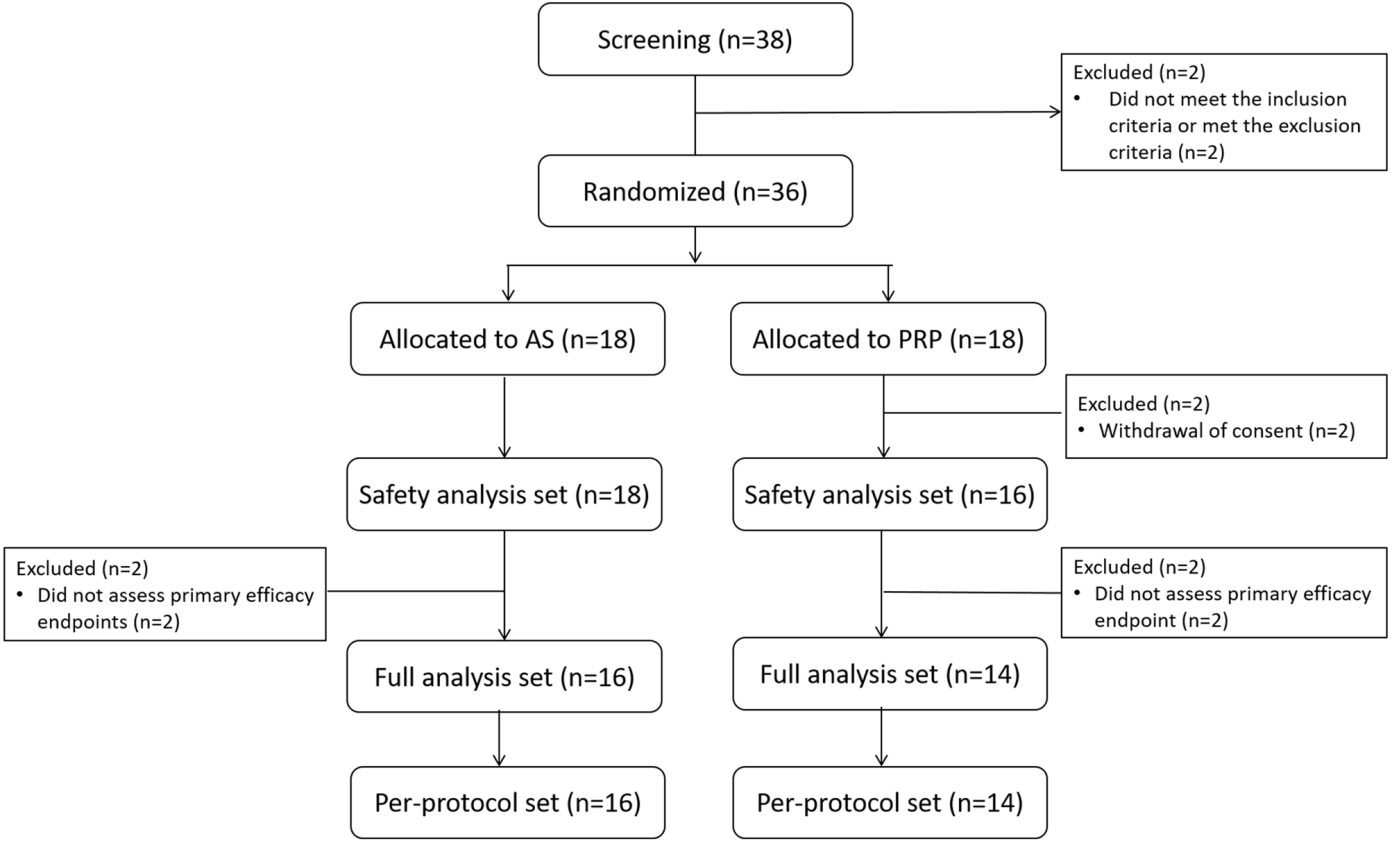
Flowchart of patient enrolment. AS, autologous serum; PRP, platelet-rich plasma.

A total of 38 patients were screened for enrolment between March 2018 and March 2019, and 36 patients were ultimately included. Four patients who did not meet the primary efficacy endpoints and two patients who withdrew their consent were excluded from the full-analysis set (FAS) (Fig. 1). All patients were female, and there was no significant age difference between the two groups (AS group, 54.56 ± 11.939 years vs. PRP group, 54.07 ± 11.283 years; p = 0.739) (Table 1).

**Table 1:** Baseline demographics in the per-protocol set.

Characteristics	Autologous serum	Platelet-rich plasma	P-value
Patients, n	16	14	
Age, years (mean ± SD)	54.56 ± 11.939	54.07 ± 11.283	0.739
Corneal staining score (mean ± SD)	3.09 ± 0.995	3.25 ± 0.844	0.518
Conjunctival staining score (mean ± SD)	3.28 ± 1.853	2.93 ± 2.054	0.487
Schirmer’s test I, mm (mean ± SD)	3.90 ± 2.784	3.65 ± 3.286	0.763
Tear break-up time, s (mean ± SD)	2.22 ± 1.008	2.29 ± 1.410	0.832
CDVA logMAR (mean ± SD)	0.059 ± 0.091	0.088 ± 0.124	0.309
OSDI score (mean ± SD)	53.133 ± 16.048	62.79 ± 22.279	0.114

SD, standard deviation; CDVA, corrected distance visual acuity; logMAR, logarithm of the minimum angle of resolution; OSDI, ocular surface disease index.

### Primary efficacy endpoints

#### Corneal staining score

**Figure 2:**
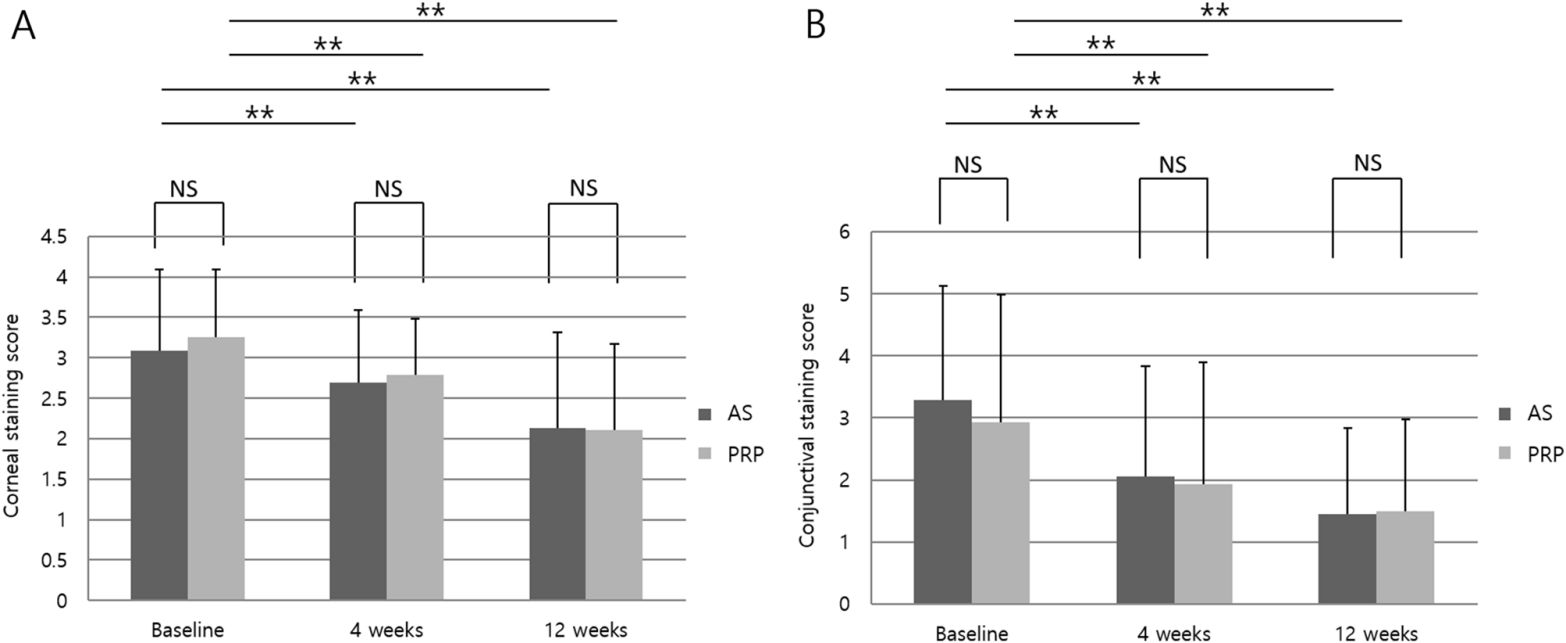
Ocular staining scores of the two groups. ** p < 0.01; error bars indicate standard deviation. (**A**) Corneal staining score; (**B**) Conjunctival staining score. AS, autologous serum; PRP, platelet-rich plasma; NS, not significant.

The baseline corneal staining score was not significantly different between the two groups (p = 0.518). Corneal staining scores improved at 12 weeks from baseline in both groups (AS group, from 3.09 ± 0.995 to 2.13 ± 1.185; p = 0.000 vs. PRP group, from 3.25 ± 0.844 to 2.23 ± 0.992; p = 0.000) (Table 2), and there was no significant difference between the two groups at 12 weeks (p = 0.952) (Fig. 2A).

**Table 2:** Change in dry eye parameters after 4 and 12 weeks of treatment.

Parameters		Autologous serum		Platelet-rich plasma		Between group P-value
		Mean ± SD	P value	Mean ± SD	P-value	
Corneal staining score	Baseline	3.09 ± 0.995		3.25 ± 0.844		0.518
	4 weeks	2.69 ± 0.896		2.79 ± 0.686		0.639
	12 weeks	2.13 ± 1.185		2.23 ± 0.992		0.952
	△ at 4 weeks	− 0.41 ± 0.665	0.002*	− 0.46 ± 0.693	0.001*	0.742
	△ at 12 weeks	− 0.97 ± 1.062	0.000*	− 1.14 ± 1.38	0.000*	0.584
Conjunctival staining score	Baseline	3.28 ± 1.853		2.93 ± 2.054		0.487
	4 weeks	2.06 ± 1.777		1.93 ± 1.961		0.782
	12 weeks	1.44 ± 1.39		1.50 ± 1.478		0.867
	△ at 4 weeks	− 1.22 ± 1.008	0.000*	− 1.00 ± 1.44	0.001*	0.494
	△ at 12 weeks	− 1.84 ± 1.417	0.000*	− 1.43 ± 1.476	0.000*	0.271
Schirmer I value	Baseline	3.9 ± 2.784		3.65 ± 3.286		0.763
	4 weeks	3.57 ± 2.897		3.96 ± 4.064		0.674
	12 weeks	3.27 ± 1.617		3.00 ± 1.766		0.558
	△ at 4 weeks	− 0.33 ± 2.057	0.325	0.31 ± 1.761	0.272	0.219
	△ at 12 weeks	− 0.63 ± 1.884	0.125	− 0.65 ± 3.212	0.418	0.976
Tear break-up time	Baseline	2.22 ± 1.008		2.29 ± 1.410		0.832
	4 weeks	3.22 ± 1.518		3.00 ± 1.122		0.533
	12 weeks	3.56 ± 1.966		3.57 ± 1.260		0.983
	△ at 4 weeks	1.00 ± 1.437	0.000*	0.71 ± 0.937	0.000*	0.360
	△ at 12 weeks	1.34 ± 1.825	0.000*	1.29 ± 1.740	0.001*	0.900
OSDI score	Baseline	53.133 ± 16.048		62.79 ± 22.279		0.114
	4 weeks	47.828 ± 16.487		56.05 ± 15.664		0.255
	12 weeks	42.451 ± 19.072		53.941 ± 18.024		0.208
	△ at 4 weeks	− 5.305 ± 15.767	0.198	− 6.727 ± 17.493	0.230	0.767
	△ at 12 weeks	− 10.682 ± 22.346	0.075	− 8.818 ± 24.314	0.254	0.843

△ = Post treatment−Baseline.

SD, standard deviation; OSDI, ocular surface disease index.

*Indicates statistical significance.

#### Conjunctival staining score

The baseline conjunctival staining score was not significantly different between the two groups (p = 0.487). Conjunctival staining scores improved at 12 weeks from baseline in both groups (AS, from 3.28 ± 1.853 to 1.44 ± 1.39; p = 0.000 vs. PRP, from 2.93 ± 2.054 to 1.5 ± 1.478; p = 0.000) (Table 2), and there was no significant difference between the two groups at 12 weeks (p = 0.867) (Fig. 2B).

### Secondary efficacy endpoints

Corneal and conjunctival staining scores at 4 weeks were compared with those at baseline. Significant reductions in the corneal staining score (p = 0.002 and p = 0.001 in the AS and PRP groups, respectively) and conjunctival staining score (p = 0.000 and p = 0.001 in the AS and PRP groups, respectively) were observed in both groups (Table 2 and Fig. 2). The Schirmer I value did not significantly change from baseline at any time point in either group (Table 2). Tear film break-up time (TBUT) improved in both groups at 4 and 12 weeks (p = 0.000 and p = 0.000 at 4 weeks; p = 0.000 and p = 0.001 at 12 weeks in the AS and PRP groups, respectively), and there was no significant difference between the two groups at 4 and 12 weeks (p = 0.533 and p = 0.983, respectively) (Table 2). The ocular surface disease index (OSDI) score did not improve from baseline at any time point in either group (Table 2). Corrected distance visual acuity did not significantly change throughout the 12 weeks in either group (data not shown). Additionally, conjunctival impression cytology (CIC) metaplasia and goblet cell density grades did not significantly change at 12 weeks from baseline in either group (Table 3).

**Table 3:** Changes in conjunctival impression cytology metaplasia and goblet cell density grades after 12 weeks.

		Autologous serum		Platelet-rich plasma		Between group P-value
		Mean ± SD	P value	Mean ± SD	P value	
Conjunctival impression cytology metaplasia grade	Baseline	0.059 ± 0.091		0.088 ± 0.124		0.309
	12 weeks	0.060 ± 0.094		0.090 ± 0.118		0.355
	△ at 12 weeks	0.090 ± 0.104		0.080 ± 0.126		0.924
Goblet cell density grade	Baseline	0.003 ± 0.082	0.831	0 ± 0.069	1.000	0.878
	12 weeks	0.028 ± 0.085	0.071	− 0.004 ± 0.077	0.802	0.144
	△ at 12 weeks	0.059 ± 0.091		0.088 ± 0.124		0.309

△ = Post treatment–Baseline.

SD, standard deviation.

*Indicates statistical significance.

### Safety endpoints

Clinical laboratory examinations, vital signs assessment, and physical examination were performed in patients who received at least one drop of either drug. Eighteen and 16 patients from the AS and PRP groups, respectively, were included in the safety analysis (Fig. 1). No adverse event was reported in either group.

## Discussion

This prospective study demonstrated that both AS and PRP eye drops are effective in improving corneal and conjunctival staining scores in primary SS DE with no significant difference at 4 and 12 weeks. In addition, TBUT improved in both groups, with no significant difference between them. Previous studies conducted on patients with non-SS DE^20^ and primary SS DE^21^ have also reported similar clinical outcomes in both groups.

The tear film has epitheliotropic factors, such as growth factors, vitamins, electrolytes, and neuropeptides that are important in the growth and migration of epithelial cells for epithelial homeostasis^22,23^. AS acts as a lubricant and supplies growth factors, cytokines, vitamins, and nutrients, similar to tears, that are crucial for ocular surface homeostasis^24^. EGF increases migration and proliferation of epithelial cells, and TGF-β decreases epithelial cell proliferation^24–26^. Fibronectin increases cell migration, and vitamin A is thought to be essential for normal epithelial cell growth^27,28^. Therefore, AS has been widely used for ocular surface disorders that do not respond to conventional commercial eye drops. Platelets are great reservoirs of growth factors, such as platelet-derived growth factor, TGF-β, EGF, and fibronectin, that are stored in α-granules and help in wound healing and tissue repair^29,30^. AS and PRP have a similar composition of growth factors and healing factors^31^; however, some studies suggested that PRP might be more advantageous than AS because the proinflammatory cytokine levels derived from leukocytes and monocytes, which are harmful to patients with immunologic disorders, are higher in AS^21,32^ and the epitheliotropic factor levels are higher in PRP than in AS^19^. TGF-β concentrations are five times higher in AS than in tears^33^. Excessively high TGF-β has been reported to suppress wound healing and promote stromal fibrosis and opacity in in-vitro studies^34,35^. Therefore, AS must be diluted to 20% to decrease the concentration of TGF-β^10^. In this study, we compared the effect of 20% AS and PRP eye drops on primary SS DE.

Some studies reported that PRP contains a 1.5 times higher level of platelets than AS^36,37^ and fewer leukocytes, which may result in an increased release of pro-inflammatory cytokines compared to AS^38^. Although growth factors do not directly correlate with platelet concentration^39^, higher levels of platelets and growth factors within the preparation might yield a better treatment outcome^21^. However, the concentrations of platelets and leukocytes in PRP and AS might vary according to the preparation method and percent concentration^40^, and no uniform preparation method exists for blood-derived eye drops; therefore, platelet and leukocyte concentrations might differ among the studies. A limitation of our study is that we did not perform a composition analysis, hence we could not elaborate on the relationship between the composition of eye drops and clinical outcomes. Further studies evaluating the exact composition of eye drops according to the selected preparation methods and percent concentrations may help improve the analyses of the clinical effect of the eye drops.

Compared with AS, PRP has a shorter preparation time using a commercial preparation kit because it does not require 2 h of clotting time. Although the preparation protocol of PRP varies according to the hospital, in our study, it was prepared with 3 min of centrifugation using a commercial preparation kit. Concerning the stability of PRP, Metheetrairut et al.^20^ reported that growth factors in PRP were stable for at least 3 months after storage at − 20 °C. They also reported an increase in some growth factors over time in PRP stored at 4 °C. They attributed the increase in growth factor concentrations to platelet activation.

The Schirmer I values did not improve in either group in our study. As the lacrimal gland is damaged in primary SS, we speculate that 12 weeks of AS or PRP treatment might not be sufficient to improve lacrimal gland function. The OSDI scores showed no improvement in both groups, but a previous study^20^ in patients with non-SS DE reported improved OSDI scores in both groups with no significant difference between the groups. Our study demonstrated that patients with SS DE had higher levels of inflammatory cytokines, such as tumor necrosis factor-α, interleukin (IL)-1β, IL-6, IL-17, and matrix metalloproteinase 9, on the ocular surface than in those with non-SS DE^41^.

This study used only AS or PRP without anti-inflammatory eye drops. Therefore, we speculate that no combined use of anti-inflammatory eye drops might be the cause of no improvement in Schirmer I and OSDI symptom scores. Further studies comparing AS and PRP with concurrent use of anti-inflammatory eye drops in primary SS DE in a real-world setting are warranted.

In this study, goblet cell metaplasia and cell density did not significantly change at 12 weeks from the baseline in the AS and PRP groups. Goblet cell metaplasia and a decrease in cell density are observed more frequently in SS DE than in non-SS DE^42,43^. Noble et al.^44^ demonstrated that treatment with 50% AS improved CIC parameters in both SS and non-SS DE, and Alio et al.^18^ demonstrated that 100% PRP improved goblet cell density in non-SS and SS DE. We speculate that a 20% concentration of AS or PRP might not be effective in goblet cell proliferation in primary SS DE.

This study had a limitation in that a population consisting of only females was included, which may have resulted in selection bias. Further studies including participants of both sexes are warranted.

In conclusion, both 20% AS and 20% PRP without anti-inflammatory eye drops are effective in improving ocular surface parameters in primary SS DE with no significant difference. Considering the shorter preparation time than that for AS, PRP eye drops might be a good alternative treatment for primary SS DE.

## Methods

### Ethics statement

This study adhered to the tenets of the Declaration of Helsinki and was approved by the institutional review board of Seoul St. Mary’s Hospital (KC17CESV0562). Written informed consent was obtained from all participants. This trial was registered in the Clinical Research Information Service (CRiS), Republic of Korea (KCT0008103; 12/01/ 2023).

### Study design and inclusion and exclusion criteria

This prospective, randomized, double-blinded clinical trial recruited patients diagnosed with primary SS with an ocular staining score of ≥ 5 points by a rheumatologist in accordance with the American College of Rheumatology and the European League Against Rheumatism (ACR-EULAR) criteria, 2016^45^. Patients with secondary SS and those using disease-modifying medications were excluded. Considering the higher prevalence of DE in women than in men (F:M = 9:1), the study population only included women^46^. Patients with the following conditions that could affect clinical DE parameters were excluded: patients who had received systemic steroid or immunosuppressive treatment within the previous 3 months; those who were pregnant or lactating; those receiving hormone treatment for menopause; patients on medication for systemic diseases, such as diabetes mellitus; those with hypertension, allergic disease, thyroid disease, depressive disorder, allergic conjunctivitis, or eyelid abnormalities; those who had undergone ocular surgery within the previous 3 months; those with ocular complications, such as ocular inflammation, infection, or trauma; patients actively using contact lenses or punctal plugs; and those using topical treatments other than artificial tears, including topical cyclosporin or steroid eye drops, within 3 months before the study period.

### Study protocol

After initial screening, a total of 36 women were enrolled and categorized into two groups using block randomization. The random number table was generated by an independent statistician using Excel (Microsoft Corp., Redmond, WA, USA). Eye drops were prepared by an independent pharmacist, and both participants and study investigators were blinded to the eye drops administered. AS and PRP eye drops were each administered to 18 patients (36 eyes). The sample size was calculated based on the rule of thumb for a pilot study suggested by Julious^47^. According to this rule, at least 12 patients were required per group; therefore, considering a dropout rate of 20%, a total of 36 patients (72 eyes) were included.

All patients used their allocated eye drops (AS or PRP) for 12 weeks at a dosage of one drop per eye six times daily in both eyes with an interval of 2 h between drops. Administration of 1–2 drops of artificial tears (sodium hyaluronate 0.1%) was allowed if needed. Participants were followed up at 4 and 12 weeks.

### Preparation of autologous serum and platelet-rich plasma eye drops

For the preparation of AS eye drops, 24 mL of peripheral venous blood was allowed to clot for 2 h at room temperature (20–25 °C) to separate the serum completely from the solid constituents. After centrifugation at 3500 g for 15 min, the serum was carefully isolated under sterile conditions in a laminar flow hood. Next, the serum was diluted to 20% (vol/vol) concentration with 0.1% (wt/vol) sodium hyaluronate preservative-free eye drops (Tearin Free; DHP Korea Co., Ltd, Seoul, Korea). Aliquots of diluted serum were stored in sterile 5 mL bottles with ultraviolet-light protection.

For the preparation of PRP eye drops, 22 mL of venous blood was withdrawn in a tube with 3 mL of 3.2% sodium citrate, and the tube was shaken slowly to prevent clotting. The blood was injected into a PRS Bio Kit (Prodizen, Seoul, Korea) through the blood inlet. The PRS Bio Kit and counter-balance kit (Prodizen, Seoul, Korea) were inserted into the centrifuge and centrifuged at 3000 g for 3 min. After centrifugation, the blood was divided into three layers (plasma, buffy coat, and red blood cells). The buffy coat was extracted using a 1-cc syringe, and plasma was extracted using a 5-cc syringe. Using a PRP connector, the buffy coat was mixed with the plasma to prepare PRP. PRP was diluted to 20% (vol/vol) concentration with 0.1% (wt/vol) sodium hyaluronate preservative-free eye drops (Tearin Free; DHP Korea Co., Ltd, Seoul, Korea).

Patients were instructed to store opened and unopened bottles of AS and PRP eye drops at 4 °C in a refrigerator and − 20 °C in a freezer, respectively.

### Clinical assessments

Corneal and conjunctival staining scores at 12 weeks were defined as the primary efficacy endpoints, and those at 4 weeks were defined as the secondary efficacy endpoints. Additionally, the Schirmer I, TBUT, and OSDI scores at 4 and 12 weeks were considered secondary efficacy endpoints. The changes in CIC metaplasia and goblet cell density grades from baseline to 12 weeks were evaluated.

The OSDI score, TBUT, corneal staining, conjunctival staining, and Schirmer I test value were assessed in order by a single investigator (S-H.C.). For TBUT evaluation, fluorescein was mixed in 15–30 mL of saline, and one drop of the solution was applied to the superotemporal conjunctiva. The average of three TBUT values was evaluated with cobalt blue light^8^.

Ocular staining scores, including corneal and conjunctival staining scores, were determined following the Sjögren’s International Collaborative Clinical Alliance registry ocular examination protocol^48^. Corneal punctate epithelial erosions (PEEs) were enumerated and scored after staining with fluorescein. Corneal scores were assigned as follows: PEE absent, zero points; 1–5 PEEs, one point; 6–30 PEEs, two points; and > 30 PEEs, three points. An additional point was awarded for each of the following cases: patches of confluent staining, staining in the pupillary area, and the presence of filaments. For conjunctival staining assessment, 1% lissamine green dye was administered at the inferior conjunctiva, and temporal and nasal bulbar conjunctival staining scores were evaluated separately, as follows: 0–9 dots, 0 points; 10–32 dots, one point; 33–100 dots, two points; and > 100 dots, three points.

For the Schirmer I test, Schirmer strips (Eagle Vision, Memphis, TN, USA) were placed in the lateral one-third of the lower eyelid without anesthesia, and the wetted length was evaluated after 5 min.

### Impression cytology

CIC was performed at least 15 min after all ocular examinations. Polyethersulfone filters (Supor 200 membrane; Pall Corporation, Port Washington, NY, USA) were applied to the superotemporal nonexposed bulbar conjunctiva after halving (13 mm × 6.5 mm). The samples were used for periodic acid–Schiff (PAS) staining.

### Periodic acid–Schiff staining

The applied filter paper was fixed for approximately 10 min in a solution of glacial acetic acid, formaldehyde, and ethyl alcohol in a 1:1:20 volume ratio^49^. PAS stain and counterstain with hematoxylin were performed^50^, and squamous metaplasia degree and goblet cell density were evaluated under a microscope. Squamous metaplasia was graded 0–3 points in accordance with Nelson’s grading system^51^ as follows: Grade 0, normal cells with normal density; Grade 1, reduced nucleocytoplasmic ratio (1:3) with decreased density; Grade 2, a nucleocytoplasmic ratio of 1:4 to 1:5 with an absence of cells; and Grade 3, a large eosinophilic cytoplasm with folded edges and pyknotic nuclei (nucleocytoplasmic ratio > 1:6) with an absence of cells plus squamous metaplasia. Thus, a higher score indicated greater metaplasia of epithelial cells. The goblet cell density grade (1–4 points) was evaluated by counting the average number of goblet cells per 100 epithelial cells in four high-power fields (HPFs), as previously described, as follows: Grade 1, > 30 goblet cells/four HPFs; Grade 2, 15–30 goblet cells/four HPFs; Grade 3, 5–15 goblet cells/four HPFs; and Grade 4, < 5 goblet cells/four HPFs^52^.

### Statistical analysis

The outcome was evaluated via a per-protocol set (PPS), FAS, and safety analyses. PPS analysis was completed in patients who finished the originally allocated treatment. FAS analysis was performed in patients who instilled at least one dose of the allocated drug with available primary efficacy endpoint data. In case of missing values, the last observation carried forward method was used for analysis. Efficacy endpoints were evaluated mainly using PPS, and FAS was also analyzed. Safety analysis was performed in all patients who received at least one dose of either drug.

Normality was tested using the Kolmogorov–Smirnov test. A paired t-test and the Wilcoxon signed-rank test were used to compare parametric and nonparametric data between the groups, respectively. Additionally, an independent t-test and the Mann–Whitney U test were used to compare parametric and nonparametric data in each group, respectively. The chi-square or Fisher’s exact test was used to evaluate the correlation of adverse events in the safety analysis. Statistical analysis was performed using Statistical Package for the Social Sciences software (version 22.0; IBM Corp., Armonk, NY, USA), and statistical significance was set at p < 0.05.

## Data Availability

The datasets generated and analyzed in the current study are available from the corresponding author on reasonable request.
